# Panduratin A from *Boesenbergia rotunda* suppresses hepatitis B virus by targeting HNF1α and synergizing with antiviral agents

**DOI:** 10.1186/s13020-025-01285-w

**Published:** 2026-01-07

**Authors:** Piyanoot Thongsri, Yongyut Pewkliang, Suparerk Borwornpinyo, Adisak Wongkajornsilp, Pakatip Ruenraroengsak, Usanarat Anurathapan, Abhasnee Sobhonslidsuk, Suradej Hongeng, Khanit Sa-ngiamsuntorn

**Affiliations:** 1 Program in Translational Medicine, Faculty of Medicine Ramathibodi Hospital, Mahidol University, Bangkok, 10400 Thailand; 2 Department of Pediatrics, Faculty of Medicine Ramathibodi Hospital, Mahidol University, 270 Ratchathewi district, Bangkok, 10400 Thailand; 3 Excellent Center for Drug Discovery, Faculty of Science, Mahidol University, Bangkok, 10400 Thailand; 4 Department of Biotechnology, Faculty of Science, Mahidol University, Bangkok, 10400 Thailand; 5 Department of Pharmacology, Faculty of Medicine Siriraj Hospital, Mahidol University, Bangkok, 10700 Thailand; 6 Department of Pharmacy, Faculty of Pharmacy, Mahidol University, Bangkok, 10400 Thailand; 7 Department of Medicine, Faculty of Medicine Ramathibodi Hospital, Mahidol University, Bangkok, 10400 Thailand; 8 Department of Biochemistry, Faculty of Pharmacy, Mahidol University, 447, Sri-Ayuthaya Road, Rajathevi District, Bangkok, 10400 Thailand; 9 Centre of Molecular Targeting and integrated Drug Development (CMT-iDD), Faculty of Pharmacy, Mahidol University, Bangkok, 10400 Thailand; 10 Ramathibodi Excellence Center for Cell and Gene Therapy, Faculty of Medicine Ramathibodi Hospital, Mahidol University, 270 Ratchathewi district, Bangkok, 10400 Thailand

**Keywords:** Hepatitis B virus, HBV, *Boesenbergia rotunda*, Fingerroot extract, Panduratin A, Pinostrobin, HNF1α, HNF4α, HBV promoter assay, shRNA knockdown, Drug combinations, Anti-HBV, Hepatocyte, 3D-liver spheroid model, imHC

## Abstract

**Background:**

*Boesenbergia rotunda* (fingerroot) is widely used in traditional medicine, and its bioactive compound panduratin A has demonstrated potent antiviral properties. However, the mechanistic basis underlying its anti-hepatitis B virus (HBV) activity remains to be fully elucidated.

**Methods:**

HBV-infected human hepatocytes (imHCs) were treated with *B. rotunda* extract, panduratin A, or pinostrobin. Intracellular HBV DNA, secreted HBsAg and HBeAg, and pregenomic RNA (pgRNA) were quantified in dose- and time-dependent experiments. Luciferase reporter assays were used to assess HBV promoter activity. The roles of HNF1α and HNF4α were evaluated by siRNA-mediated knockdown and ectopic gene expression. Drug interaction studies were performed using the KDM5 inhibitor GS-5801 and the capsid assembly modulator NVR-3778. A 3D liver spheroid model was used to validate antiviral effects on HBV DNA and cccDNA. Gene interaction network analysis was conducted to identify central regulatory pathways.

**Results:**

*B. rotunda* extract, panduratin A, and pinostrobin significantly suppressed intracellular HBV DNA, HBsAg, HBeAg, and pgRNA. Panduratin A exhibited the strongest antiviral activity and inhibited preS1, preS2, and core promoter activities. Panduratin A markedly downregulated HNF1α expression, with only modest effects on HNF4α. Knockdown of HNF1α significantly reduced the antiviral efficacy of panduratin A, whereas ectopic HNF1α expression rescued its inhibitory effects. Co-treatment with GS-5801 produced synergistic activity, and combination with NVR-3778 yielded additive antiviral effects. In 3D liver spheroids, panduratin A reduced intracellular HBV DNA and cccDNA with minimal cytotoxicity. Network analysis further identified HNF1α as a key regulatory node modulated by panduratin A.

**Conclusion:**

Panduratin A is a potent anti-HBV compound that acts primarily through HNF1α-dependent suppression of HBV transcription and replication. Its efficacy in combination therapy and in 3D liver models highlights its potential as a promising candidate for future HBV treatment strategies.

**Graphical Abstract:**

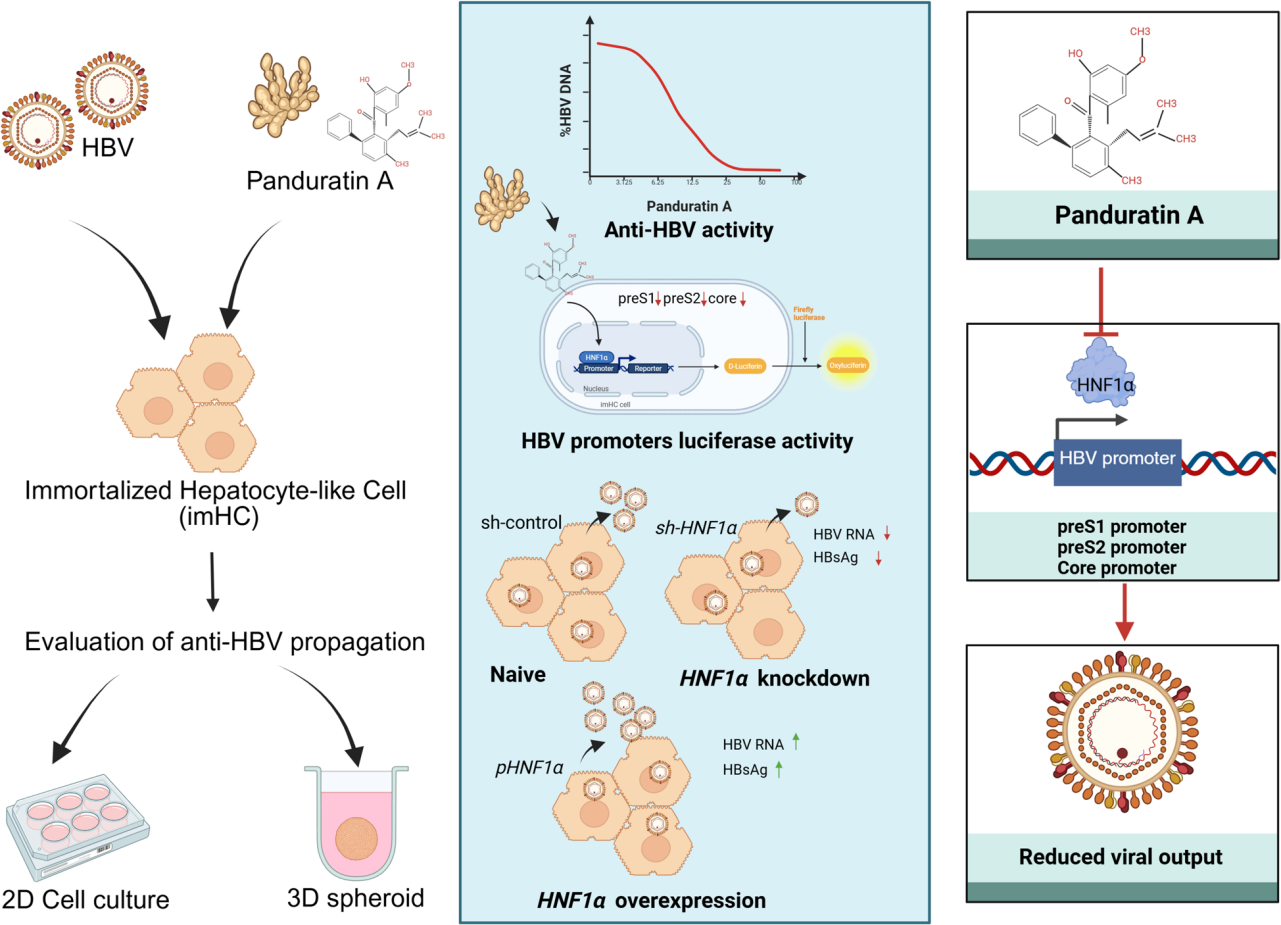

**Supplementary Information:**

The online version contains supplementary material available at 10.1186/s13020-025-01285-w.

## Introduction

Hepatitis B virus (HBV) infection has been a significant global health burden since its discovery in 1989. Currently, the global HBV infection rate stands at approximately 3.5%, with over 250 million individuals suffering from chronic infection, with 1.2 million new cases reported annually [1, 2]. Notably, nearly 90% of infants or children infected perinatally develop chronic HBV infection, whereas most adults with acute HBV infection achieve spontaneous clearance, according to clinical data [3]. Recent data indicate that HBV-related infections account for approximately 887,000 deaths annually [4]. While most patients recover from acute HBV infection, some develop chronic infection and hepatocellular carcinoma (HCC). Treating chronic HBV infection remains challenging due to the persistence of covalently closed circular DNA (cccDNA) within hepatocytes, which serves as a template for viral replication and cannot be eliminated by current antiviral therapies. The persistence of HBV DNA and the chronicity of the infection contributes to the progression of severe liver diseases, including fibrosis and cirrhosis, eventually leading to HCC.

The hepatitis B virus (HBV) vaccine is highly effective and safe, significantly reducing the incidence of new HBV infections. However, a small proportion of vaccinated individuals may fail to develop adequate immunity against the virus [5]. The rising number of HBV cases in some countries can be attributed to factors such as high rates of injection drug use, unprotected sexual activity, and limited vaccine availability or accessibility in certain regions [5, 6]. HBV infection is particularly prevalent in sub-Saharan Africa and Southeast Asia, where approximately 5–7% of the population is affected [7]. Current standard treatment include nucleos(t)ide analogs (NAs), such as lamivudine, entecavir, tenofovir, telbivudine, and adefovir [8]. NAs inhibited reverse transcriptase enzyme by incorporating into DNA strand, causing chain termination. However, NA therapies rarely eradicate HBV persistence because cccDNA remained in the hepatocyte. Over the last decade, various drugs, including direct-acting antivirals (DAAs) which repress viral RNA or protein synthesis, and host-targeted antivirals such as HBV entry inhibitors or immunomodulators, have entered phase I and II clinical trials [9]. DAAs in the trials included core protein inhibitors, siRNAs, pol/RT inhibitors and HBsAg secretion inhibitors [9]. Additionally, the emergence of drug-resistant HBV strains presents a significant challenge. Future HBV therapies should aim not only to inhibit the virus directly but also to achieve a functional cure. A primary target for novel treatments is covalently closed circular DNA (cccDNA), which is crucial for HBV persistence.

HBV is an enveloped virus with a partially double-stranded relaxed circular DNA (rcDNA) genome, classified within the *Hepadnaviridae* family. Its genome is approximately 3.2 kilobases in length and contains HBV polymerase within the nucleocapsid. The HBV genome consists of four overlapping open reading frames (ORFs): pre-core/core (preC/C), polymerase (P), pre-surface/surface (preS/S), and X. These ORFs are transcribed into polyadenylated RNAs of 3.5, 2.4, 2.1, and 0.7 kilobases, respectively [10, 11]. The rcDNA of HBV is repaired within host cells to form cccDNA, which serves as the template for transcription and replication in the nuclei of infected hepatocytes. Due to the existence of cccDNA in the nucleus, viral clearance remains difficult, resulting in continued HBV replication and frequent relapse [12]. The transcription of HBV RNA plays a crucial role in the production of both new and recycled cccDNA. This process requires multiple nuclear factors to facilitate HBV RNA synthesis. In recent years, liver-enriched transcription factors (LETFs) have emerged as promising targets for inhibiting HBV RNA transcription. Notable LETFs include hepatocyte nuclear factors (HNFs), the farnesoid X receptor (FXR), and peroxisome proliferator-activated receptor γ coactivator-1 α (PGC1α) [13]. Several HNFs, such as HNF1α, HNF4α, HNF3β, and HNF6, are essential for HBV transcription and replication [14]. HNF1α interacts with key HBV regulatory elements, including the core, pre-S1, and HBx promoters, as well as the cccDNA Enhancer II. Similarly, HNF4α binds to the preS2 and core promoters [15]. Targeting these nuclear transcription factors involved in HBV transcription offers a novel therapeutic strategy to eliminate HBV infection.

*Boesenbergia rotunda* (L.) Mansf., also known as *Kaempferia pandurata* Roxb. or *Boesenbergia rotunda*, and commonly referred to as fingerroot, belongs to the ginger family (Zingiberaceae). This plant is native to Southeast Asia and has been extensively used in traditional medicine, including Traditional Chinese Medicine (TCM), due to its numerous health benefits. In TCM, herbal remedies derived from rhizomatous plants like *Boesenbergia rotunda* have long been valued for their ability to support digestive health, resolve inflammation, and enhance vital energy (Qi). Several studies have explored the antiviral properties of *B. rotunda*. Notably, a study demonstrated that *Boesenbergia rotunda* and its key bioactive compound, panduratin A, exhibited potent inhibitory activity against SARS-CoV-2, the virus responsible for the COVID-19 pandemic that emerged in 2019 [16]. Panduratin A showed strong antiviral effects against SARS-CoV-2 in human cardiomyocytes while also offering cardioprotective benefits [17]. Additionally, *B. rotunda* has shown anti-HIV protease activity [18]. Its hepatoprotective potential has also been reported in animal models, particularly in rats [19, 20]. Collectively, these findings suggest that *B. rotunda* and its active compound, panduratin A, possess significant antiviral properties and offer protective effects against liver damage, supporting its continued relevance in the context of integrative approaches combining modern pharmacology and traditional practices such as Chinese medicine.

In this study, the crude extract of *Boesenbergia rotunda* and its purified compounds, panduratin A and pinostrobin, demonstrated inhibitory effects on the replication of HBV. These compounds effectively decreased HBV viral load, intracellular HBV DNA, and the levels of HBV proteins, including HBsAg, HBeAg, and HBcAg, comparable to direct-acting antiviral drugs (DAAs). Particularly, panduratin A exhibited superior anti-HBV activity, reducing HBV DNA levels more effectively than entecavir. The inhibitory effects of panduratin A involved in downregulating HNF1α, thereby inhibiting HBV transcription and replication through modulation of the HNF1α axis.

## Material and methods

### Reagents and chemicals

The *Boesenbergia rotunda* extract was provided by the Excellent Center for Drug Discovery (ECDD), Faculty of Science, Mahidol University, Thailand, as previously described [16]. *B. rotunda* extract, panduratin A (CAS: 89837–52-5, PhytoLab, Germany), and pinostrobin (CAS: 38790, Sigma, MO, USA) were dissolved in dimethyl sulfoxide (DMSO) to prepare 10 mg/mL and 10 µM stock solutions, which were stored at − 20 °C until use.

### Cell culture

Immortalized hepatocyte-like cells (imHC) were maintained in a 1:1 mixture of DMEM/F12 (Hyclone). HepG2.2.15 cell was cultured in high-glucose DMEM. All media were supplemented with 10% fetal bovine serum (Hyclone), 100 U/mL penicillin, 100 µg/mL streptomycin (Invitrogen, USA), and 2 mM L-glutamine (GlutaMAX, Gibco, Thermo Fisher Scientific, MA, USA) at 37 °C in a humidified atmosphere containing 5% CO₂. imHC cells were cultured in complete medium supplemented with 2% DMSO for two weeks before HBV inoculation [21, 22].

### Production of cell culture-derived HBV (HBV_cc_)

HepG2.2.15 cells were maintained in complete DMEM supplemented with 250 µg/mL geneticin (G8168, Sigma, MO). HepG2.2.15-derived HBV particles (HBVcc) were harvested every 3–4 days and stored at 4 °C. The conditioned medium was clarified by centrifugation at 1500×*g* for 30 min. The supernatant was mixed with a lentivirus concentrator (Clontech, Takara Bio, CA) at a 3:1 ratio, gently mixed, and incubated at 4 °C overnight. The HBVcc pellet was collected by centrifugation at 1500×*g* for 45 min at 4 °C. The HBV particles were resuspended in fetal bovine serum (FBS) and stored at − 80 °C until use [23]. HBV titers were quantified using absolute real-time PCR (qPCR) to calculate genome equivalents per milliliter (GE/mL), with a known amount of HBV 1.3-mer WT plasmid (Addgene plasmid #65459) as a standard reference.

### Cytotoxicity assay of *B. rotunda* extract and its bioactive compounds

The imHC cells were seeded onto 96-well plates at a density of 2 × 10^4^ cells per well for 24 h and then treated with 0–100 µg/mL *B. rotunda* extract, 0–100 µM panduratin A, or 0–100 µM pinostrobin for 48 h. Cell viability was assessed using the MTT assay. Briefly, treated imHC cells were incubated in a medium containing MTT solution for 3 h. The medium was removed, and DMSO was added to dissolve the formazan crystals. Absorbance was measured at 570 nm with a reference wavelength of 650 nm using a microplate reader (Tecan, Switzerland).

### The inhibitory effects of *B. rotunda* extract and its bioactive compounds on HBV replication and life cycle

For HBV infection, differentiated imHC (d-imHC) cells were inoculated with HBVcc at an MOI of 100 for 16 h before treatment with bioactive compounds. Infected d-imHC cells were treated with 0–100 µg/mL *B. rotunda* extract, 0–12 µM panduratin A, or 0–12 µM pinostrobin for 10 days. For the HBV replicon system, d-imHC cells were transfected with the HBV 1.3-mer WT replicon and incubated for 18 h before treatment with 0–100 µg/mL *B. rotunda* extract, 0–12 µM panduratin A, or 0–12 µM pinostrobin for 10 days. Direct-acting antivirals (DAAs) at 10 µM, including entecavir (ETV), lamivudine (LMV), tenofovir alafenamide (TAF), and tenofovir disoproxil fumarate (TDF), were used as positive controls. Hepatocyte pellets and conditioned media were collected to evaluate intracellular HBV DNA and HBV viral load, respectively.

### The detection of HBV DNA and host gene expressions by quantitative real-time PCR

Intracellular and extracellular HBV DNA were extracted from infected hepatocytes and conditioned medium using a DNA extraction kit (NucleoSpin, MN, Düren, Germany). A total of 50 ng of total cellular DNA or HBV DNA sample was mixed with specific primers (HBV DNA) and KAPA SYBR FAST qRT-PCR Kit solution (Kapa Biosystems, MA). qPCR amplification was performed using a CFX96 Touch Real-Time PCR Detection System (Bio-Rad, CA). PRNP was used as the reference gene for intracellular HBV DNA. Extracellular HBV DNA was used to determine HBV viral load, with plasmid HBV 1.3-mer WT replicon serving as a standard curve calibrator (Addgene plasmid #65459). For host gene expression analysis, total RNA was extracted using the GE Healthcare illustra™ RNAspin Mini Isolation Kit (GE Healthcare, IL). A total of 4 µL of total RNA was reverse transcribed into cDNA using the ImProm-II™ Reverse Transcription System kit (Promega, WI). Gene expression levels of hepatocyte host factors, including HNF1α, HNF4α, PPARα, C/EBPα, FoxO4, and HBV genes (pgRNA, HBV RNA and HBx) were analyzed using specific primers (Table S1), with GAPDH serving as the reference gene. The qPCR cycling conditions were as follows: 95 °C for 5 s, followed by 40 cycles of 95 °C for 15 s, 60 °C for 4 s, 72 °C for 25 s, and detection at 88 °C for 2 s after each cycle.

### Detection of HBV cccDNA by exonuclease treatment and PCR

Infected hepatocytes were harvested from 6-well plates using 0.125% trypsin–EDTA. For covalently closed circular DNA (cccDNA) detection, relaxed circular DNA was removed by exonuclease digestion following plasmid DNA extraction. The cccDNA was then amplified using specific primers (Table S1), yielding a 579 bp product that spans the gap and nick regions of the HBV relaxed circular DNA. The PRNP gene was used as an internal control. PCR was performed under optimized conditions: an initial denaturation at 95 °C for 3 min, followed by 40 cycles of 95 °C for 3 s and 60 °C for 30 s. The amplified cccDNA products were confirmed by gel electrophoresis.

### Quantification of HBV covalently closed circular DNA (cccDNA) by droplet digital PCR (ddPCR)

HBV covalently closed circular DNA (cccDNA) was quantified using droplet digital polymerase chain reaction (ddPCR) [24]. Total DNA was extracted from infected-imHC cells using the NucleoSpin^®^ DNA Miniprep Kit (Macherey–Nagel, Germany). One microgram of DNA was digested with FastDigest EcoRI (Thermo Fisher Scientific, USA), an enzyme that specifically cleaves the HBV subtype ayw genome without affecting the target region of the reference amplicon. Each 25 µL ddPCR reaction contained 12.5 µL of 2 × ddPCR Supermix for Probes (Bio-Rad, USA), 900 nM of each forward and reverse primer, 250 nM of hydrolysis probe for both HBV cccDNA and the reference gene RPP30 (primer and probe sequences are shown in Table S1), and 1 µL of digested DNA. For droplet generation, 20 µL of the reaction mixture and 70 µL of Droplet Generation Oil were loaded into DG8™ cartridges (Bio-Rad), sealed with DG8™ gaskets, and processed using a QX200™ Droplet Generator (Bio-Rad). Approximately 40 µL of droplets were transferred to a 96-well PCR plate and heat-sealed with pierceable foil at 180 °C for 5 s. Thermal cycling was performed on a T100™ Thermal Cycler (Bio-Rad) under the following conditions: 95 °C for 10 min; 45 cycles of 94 °C for 30 s and 58 °C for 1 min; followed by enzyme deactivation at 98 °C for 10 min. After amplification, droplets were read using a QX200™ Droplet Reader, and data were analyzed with QuantaSoft™ software (Bio-Rad). The HBV cccDNA copy number variation (CNV) was calculated using the following formula: CNV = 2 × (concentration of cccDNA / concentration of RPP30).

### Quantification of HBsAg and HBeAg by sandwich ELISA

Secreted hepatitis B surface antigen (HBsAg) and hepatitis B e antigen (HBeAg) levels in conditioned medium were measured using ELISA detection kits (KA0286 and KA0290, Abnova, USA) according to the manufacturer’s instructions. Absorbance was measured at 450 nm using a CLARIOstar Plus Microplate Reader (BMG Labtech, Germany).

### Quantification of HBV core and HBx proteins by flow cytometry

Hepatocytes were harvested and fixed with 4% paraformaldehyde for 20 min. After two washes, cells were permeabilized with 0.2% Triton-X 100 for 15 min and washed twice with PBS. Cells were incubated with an anti-HBV core antibody (ab8637, Abcam, UK) or anti-Hepatitis B Virus X antigen antibody (ab235, Abcam, UK) at a 1:200 dilution for 1 h at 4 °C, washed three times, and stained with an Alexa Fluor 488-conjugated secondary antibody (1:500 dilution) for 1 h at 4 °C. Finally, cells were resuspended in 1% FBS in PBS and analyzed using a Beckman Coulter CytoFLEX S flow cytometer.

### The inhibitory effect of *B. rotunda* extract, panduratin A, and drugs on HBV promoter activity using a luciferase assay

imHC cells were seeded onto a 96-well plate (1.0 × 10^4^ cells/well) and maintained in a CO_2_ incubator overnight. Cells were transfected with 150 ng/well of each HBV promoter plasmid, including the HBV core promoter pHBV-Luc (Addgene plasmid #71414), HBV S1 promoter pHBV-S1-Luc (Addgene plasmid #71416), HBV S2 promoter pHBV-S2-Luc (Addgene plasmid #71417), and HBV X promoter pHBV-X/EnhI-Luc (Addgene plasmid #71418), using Lipofectamine 3000 transfection reagent (Invitrogen, MA). *B. rotunda* extract, panduratin A, or HBV drugs were added 24 h post-transfection. Luciferase activity was measured using the Dual-Glo® Luciferase Assay System kit (E2620, Promega, WI) and detected using the CLARIOstar Plus plate reader (BMG Labtech, Germany).

### Immunofluorescence assay (IFA)

imHC and HBV-infected imHC cells were treated with herbal compounds or commercial drugs for 7 days. Cells were fixed with 4% (w/v) paraformaldehyde, permeabilized with 0.2% (v/v) Triton X-100 in PBS, and incubated with anti-HBcAg antibody (ab8637, Abcam, UK), anti-preS2 Ag antibody (ab8635, Abcam, UK), HNF4α (ab41898, Abcam, UK), and HNF1α (ab96777, Abcam, UK) at a 1:200 dilution at 4 ºC overnight. After washing three times, cells were incubated with Alexa Fluor 568-conjugated goat anti-mouse IgG and Alexa Fluor 488-conjugated goat anti-rabbit IgG (A11004, A11034, Life Technologies, USA) for 1 h. Cells were washed three times, incubated with DAPI (Thermo Fisher Scientific, USA) for 10 min, and visualized using a Nikon Eclipse E800 microscope (Nikon, Japan).

### Determination of protein expression level by Western Blot

Treated cells were harvested from 6-well plates using 0.125% trypsin–EDTA. Cellular protein was extracted from cell pellets using 100 μL of RIPA buffer (Merck, MA), and total protein concentration was quantified using the BCA assay. Proteins were separated by SDS-PAGE and transferred onto 0.22 µm polyvinylidene difluoride (PVDF) membranes (IPVH00010, Millipore, MA). The membranes were blocked with 5% skim milk in 0.5% TBS-T buffer for 1 h, followed by overnight incubation with primary antibodies at a 1:500 dilution. The primary antibodies included anti-GAPDH, HNF4α, and HNF1α. After washing twice for 5 min, the membranes were incubated with HRP-conjugated anti-mouse or anti-rabbit secondary antibodies (1:1000 dilution) for 1 h. Protein detection was carried out using HRP chemiluminescence substrates (WBLUR0500, Millipore, USA) with the ChemiDoc MP Imaging System (Bio-Rad, CA). GAPDH was used to normalize protein levels of interest.

### Liver spheroid culture

imHC cells were cultured at a density of 1 × 10^4^ cells/well in 96-well Black/Clear Round Bottom Ultra-Low Attachment plates (Cat No. 4515, Corning, USA) for 3 days. imHC spheroids were either infected with HBVcc at a multiplicity of infection (MOI) of 100 overnight or transfected with the HBV 1.3-mer WT plasmid (5 ng/well) for 4 h. After incubation, imHC cells were maintained in complete William’s medium. On day 6 post-incubation, the infected/transfected cells were treated with panduratin A or classical HBV drugs diluted in complete William’s medium. The culture medium was changed every 2–3 days until day 10. imHC spheroids were analyzed using the EVOS™ M7000 Imaging System (Thermo Fisher Scientific, MA).

### Statistical analysis

All experiments were performed in triplicate, and the results are expressed as the mean ± standard deviation (SD). Statistical analyses were conducted using GraphPad Prism 9.5 software. Differences between two groups were assessed using an unpaired two-tailed Student’s *t*-test. For comparisons involving multiple groups, one-way analysis of variance (ANOVA) was applied, followed by post-hoc testing. Dunnett’s test was used for comparisons against a single reference group, while Tukey’s Honest Significant Difference (HSD) test was employed for pairwise comparisons among multiple groups. A p-value of less than 0.05 was considered statistically significant.

## Results

### *Boesenbergia rotunda* extract and its active compounds inhibited HBV DNA replication and viral propagation

**Fig. 1:**
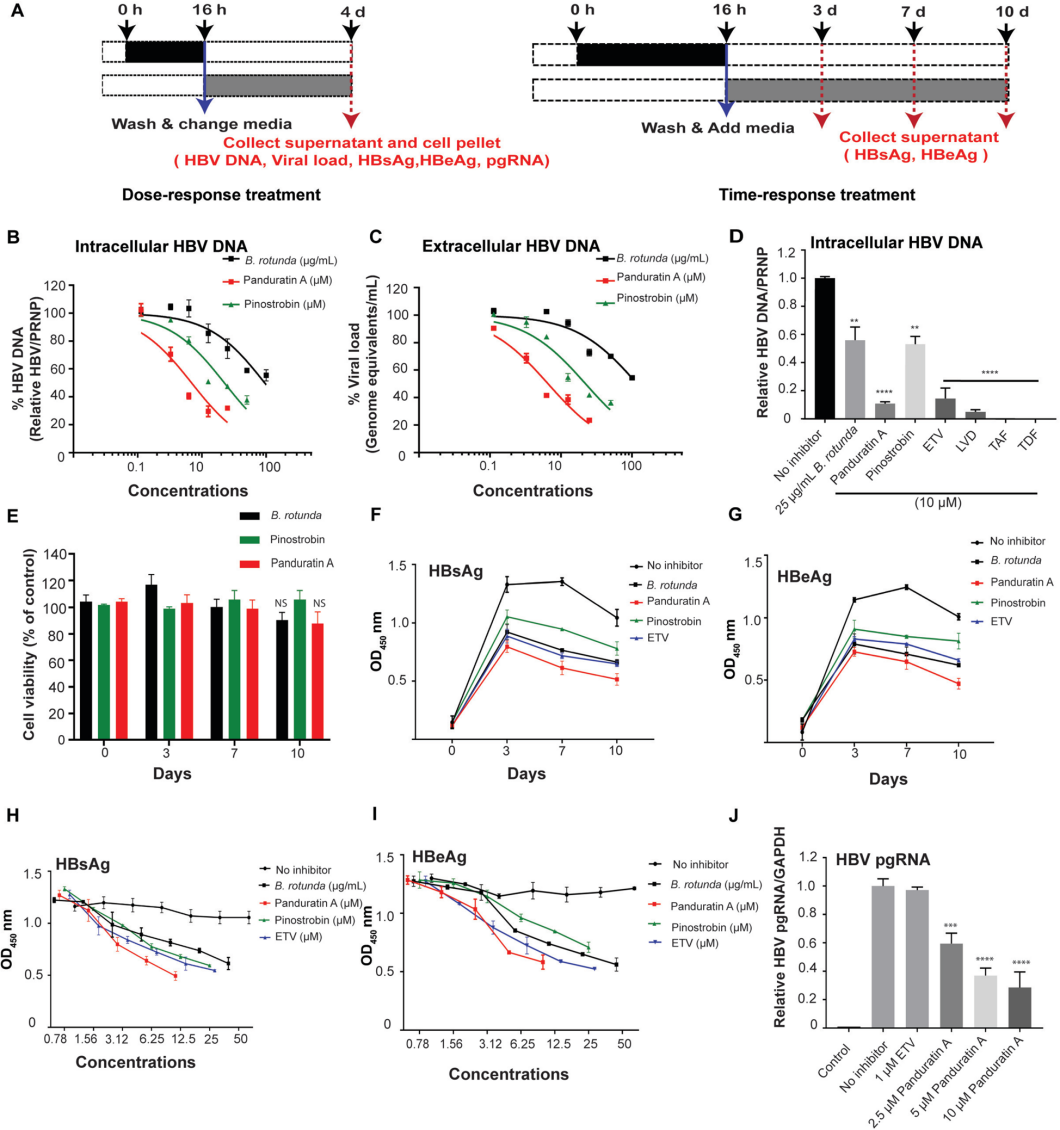
*Boesenbergia rotunda* extract, panduratin A, and pinostrobin inhibited HBV DNA replication and reduced HBsAg, HBeAg, and pgRNA levels. Schematic of the experimental design (**A**). *B. rotunda* extract, panduratin A, and pinostrobin reduced HBV DNA replication and viral load in a dose-dependent manner (**B**, **C**). These compounds effectively inhibited HBV DNA production compared to classical anti-HBV drugs, including 10 µM ETV, LMV, TAF and TDF (**D**). Cytotoxic effects of *B. rotunda extract*, panduratin A, and pinostrobin were evaluated over a 10-day period (**E**). HBsAg and HBeAg levels in conditioned medium were measured by ELISA on days 0, 3, 7, and 10 following treatment with *B. rotunda* extract, panduratin A, pinostrobin, and entecavir (**F**, **G**). HBsAg and HBeAg levels on day 4 were assessed by ELISA after treatment with *B. rotunda* extract (0.78–50 μg/mL), panduratin A (0.78–12.5 μM), pinostrobin, and entecavir (**H**, **I**). Panduratin A reduced HBV pgRNA levels in a dose-dependent manner, as determined by real-time qPCR, compared to ETV (**J**). N = 3; Data were presented as mean ± SD. *, **, ***, and **** indicate statistical significance at p values < 0.05, < 0.01, < 0.001, and < 0.0001, respectively

We initially evaluated the cytotoxicity of *Boesenbergia rotunda* (*B. rotunda*) extract (0–100 µg/mL) and its bioactive compounds, panduratin A (0–100 µM; Fig. S1A) and pinostrobin (0–100 µM; Fig. S1B), in imHC cells. The observed 50% cytotoxic concentrations (CC_50_) were 72.30 µg/mL for *B. rotunda* extract, 15.91 µM for panduratin A, and > 100 µM for pinostrobin (Fig. S1C–E). Next, infected d-imHC cells were treated with 0–50 µg/mL *B. rotunda* extract, 0–12 µM panduratin A, or 0–12 µM pinostrobin to assess dose- and time-dependent effects on HBV DNA levels on day 4 and day 3, 7, 10 post-treatment, respectively (Fig. 1A).

For intracellular HBV DNA, *B. rotunda* extract, panduratin A, and pinostrobin reduced replication by 50% (IC_50_) at > 50 µg/mL, 5.41 µM, and 10.56 µM, respectively (Fig. 1B). Similarly, the compounds decreased HBV DNA progeny by 50% (IC_50_) at > 50 µg/mL, 5.23 µM, and 12.31 µM, respectively, in a dose-dependent manner (Fig. 1C). To compare efficacy, these bioactive compounds were evaluated alongside direct-acting antiviral (DAA) drugs, ETV, LMV, TAF and TDF, all tested at 10 µM. On day 4 post-infection, treatment with 25 µg/mL *B. rotunda* extract, 10 µM panduratin A, and 10 µM pinostrobin decreased intracellular HBV DNA by 57.28%, 89.10%, and 46.87%, respectively. By comparison, ETV, LMV, TAF, and TDF decreased intracellular HBV DNA by 89.1%, 94.9%, 99%, and 99%, respectively (Fig. 1D).

The kinetics of HBV antigen reduction were further evaluated. Cytotoxicity assays of 10 µg/mL *B. rotunda* extract, 10 µM pinostrobin and 5 µM panduratin demonstrated no to low toxicity over a 10-day period (Fig. 1E). Treatment with 10 µg/mL *B. rotunda* extract or 5 µM panduratin A decreased secretory HBsAg levels by 60–70% between days 3 and 7 post-infection (dpi). In contrast, pinostrobin at 10 µM caused a 30% decrease in secretory HBsAg, while 1 µM ETV achieved a 50% decrease (Fig. 1F). Similarly, *B. rotunda* extract, panduratin A, and pinostrobin decreased secretory HBeAg by 60–70% in a dose-dependent manner (Fig. 1G). A dose–response analysis revealed that 0.78–50 µg/mL *B. rotunda* extract, 0.78–12.5 µM panduratin A, and 0.78–12.5 µM pinostrobin decreased HBsAg levels by 20–80% and HBeAg levels by 20–70% (Fig. 1H, I). Furthermore, 2.5–10 µM panduratin A significantly decreased HBV pregenomic RNA (pgRNA) levels in a dose-dependent manner, whereas 1 µM ETV treatment showed no inhibitory effect. (Fig. 1J).

Overall, these findings indicated that *B. rotunda* extract, pinostrobin, and particularly panduratin A exhibited strong potential for development as anti-HBV agents. Owing to its robust anti-HBV activity, panduratin A was selected for further mechanistic studies.

### Panduratin A decreased the expressions of HBcAg and HBsAg through promoter regulation

**Fig. 2:**
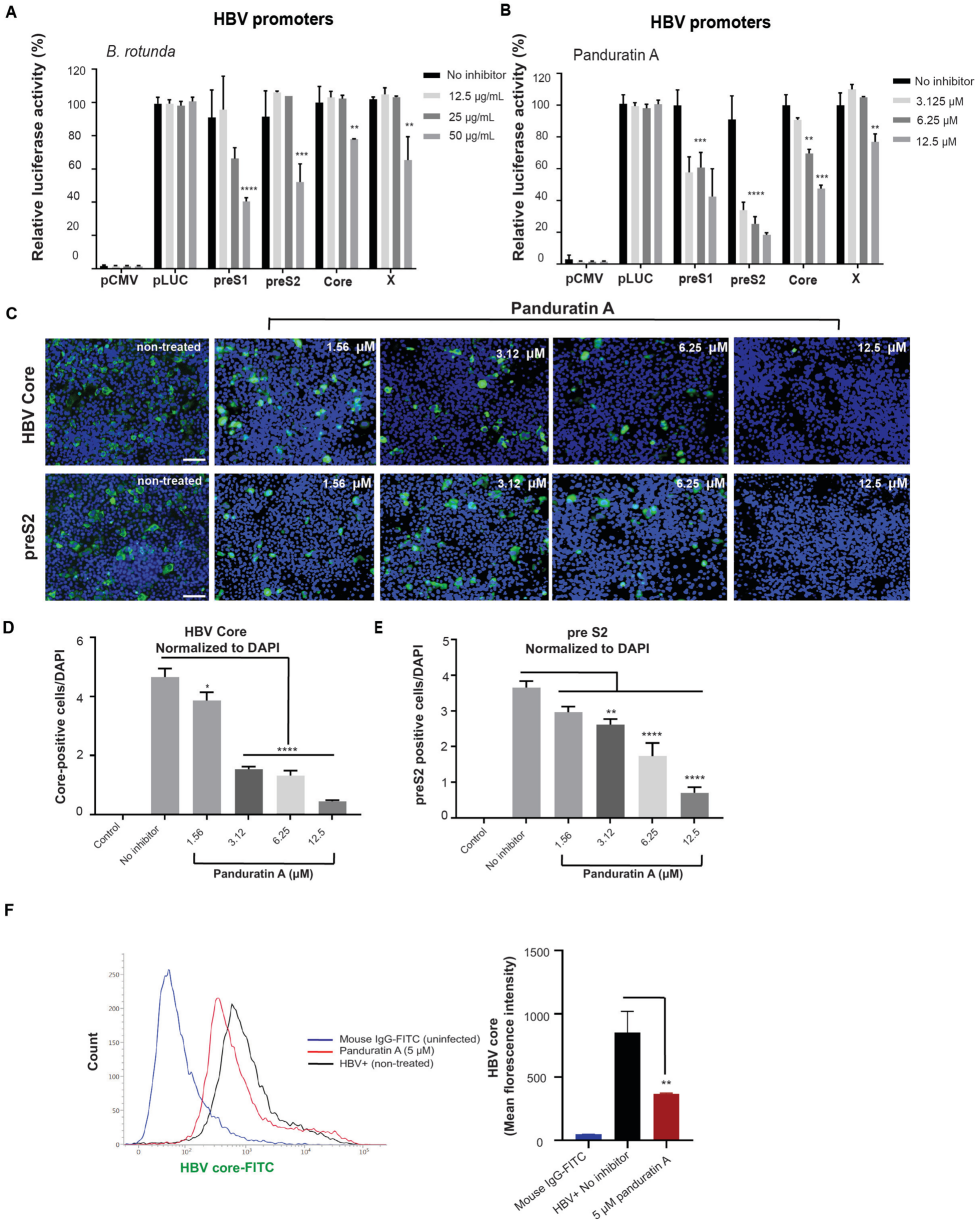
*Boesenbergia rotunda* extract and panduratin A reduced HBcAg and HBsAg protein levels by modulating HBV promoter activity. imHC cells were transfected with an HBV promoter-containing plasmid for 4 h, followed by treatment with *B. rotunda* extract or panduratin A for 2 days. *B. rotunda* extract (0–50 µg/mL) and panduratin A (0–12.5 µM) reduced preS1, preS2, C, and X HBV promoter activities, as quantified by a luciferase assay (**A**, **B**). Panduratin A decreased HBV infectivity in d-imHC cells on day 7, as determined by immunofluorescence assay (IFA) using HBcAg and preS2 antibodies (scale bar: 100 μm) (**C**, **D**). Relative fluorescence intensity per cell (Green/DAPI) was analyzed (**E**). Flow cytometry analysis showed that panduratin A suppressed HBV core protein expression (**F**). N = 3; Data were presented as mean ± SD. *, **, ***, and **** indicate statistical significance at p values < 0.05, < 0.01, < 0.001, and < 0.0001, respectively

To investigate the mechanisms by which *B. rotunda* extract and panduratin A inhibit HBV DNA and pgRNA production, we examined their effects on HBV promoter activity. Since fingerroot extract strongly reduced pgRNA transcription, we assessed the impact of 0–50 µg/mL *B. rotunda* extract and 0–12.5 µM panduratin A on HBV promoters. imHC cells were transfected with HBV promoter-luciferase plasmids, including pHBV-S1-Luc (preS1), pHBV-S2-Luc (preS2), pHBV-Luc (core), pHBV-X/EnhI-Luc (X promoter), pLuc (luciferase control) or pCMV (empty plasmid) for 24 h before treatment. On day 2 post-treatment, HBV promoter activities were measured using a luciferase-based reporter assay. Luminescence analysis showed that 50 µg/mL *B. rotunda* extract suppressed preS1 and preS2 promoter activities by more than 50%, while it moderately reduced X and core promoter activities by approximately 25% and 40%, respectively (Fig. 2A). For the purified compound, 3.12–12.5 µM panduratin A significantly suppressed preS1, preS2, and core promoter activities by 40–60%, 60–80%, and 25–50%, respectively. HBV X promoter activity decreased by 30% following treatment with 12.5 µM panduratin A (Fig. 2B), while entecavir treatment had no effect on HBV promoter activity (Fig. S1F). HBV core and surface antigen (HBcAg and HBsAg) levels were detected in infected d-imHC cells using immunofluorescence assays, which demonstrated decreased HBcAg and preS2 expression at 7 dpi (Fig. 2C). Quantitative fluorescence image analysis confirmed that 1.56–12.5 µM panduratin A significantly reduced HBcAg by 20–90% (Fig. 2D) and preS2 (HBsAg) by 25–75% (Fig. 2E) compared with entecavir (Fig. S1G). Additionally, flow cytometry histograms showed that the percentage of HBcAg-positive hepatocytes decreased following treatment with 5 µM panduratin A for 4 days (Fig. 2F). The effect of 0–10 µM panduratin A on HBx expression was examined in both HBV-infected imHC cells and imHC cells with ectopic HBx expression. Panduratin A slightly reduced HBx mRNA expression by approximately 15% (Fig. S2A) and 17% (Fig. S2B), respectively. However, panduratin A did not reduce HBx protein levels (Fig. S2C). Collectively, these findings suggest that panduratin A inhibits HBV promoter activity by modulating preS1, preS2, and core gene transcription, thereby interfering with the production of essential viral proteins.

### Downregulation of HNF1α primarily mediated the anti-HBV activity of *B. rotunda* and panduratin A

**Fig. 3:**
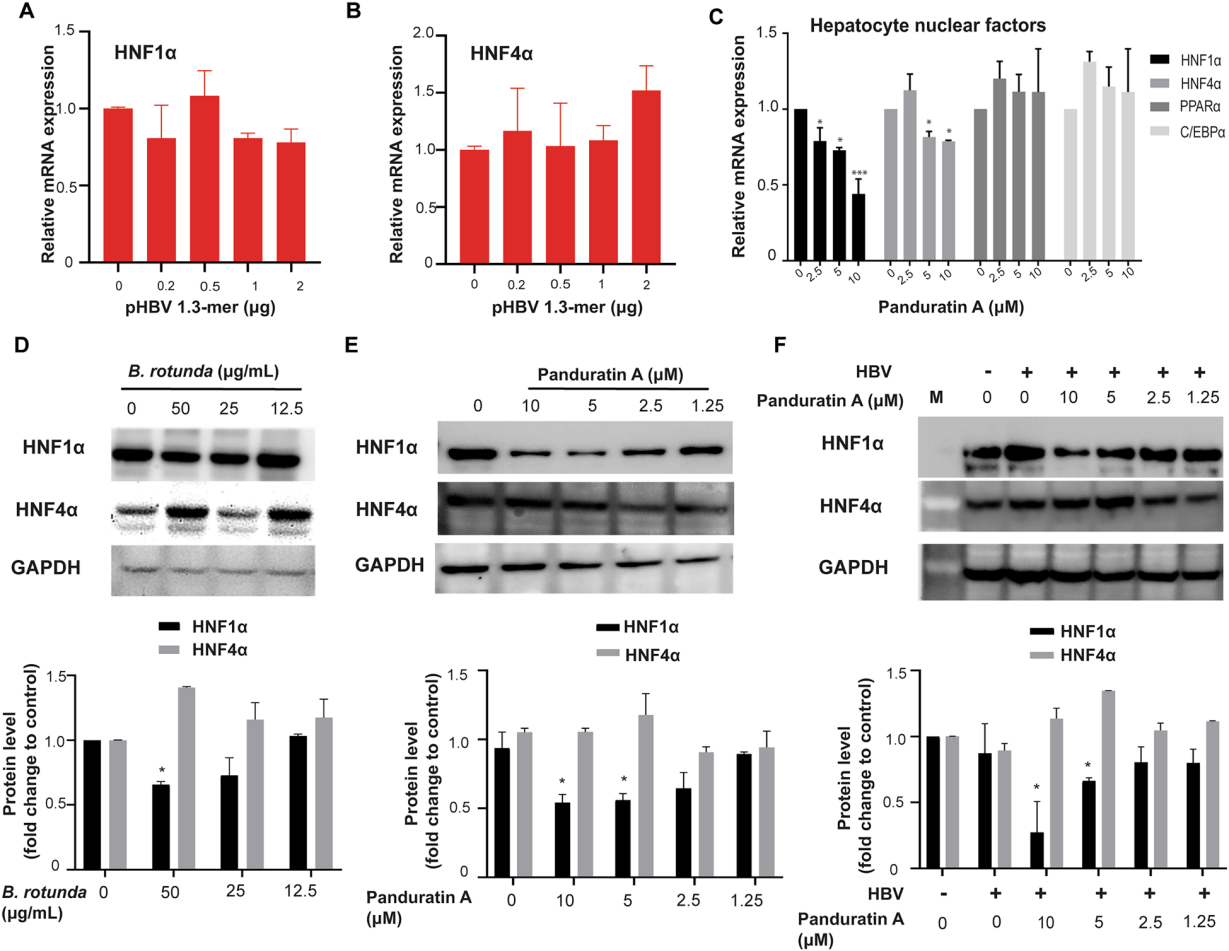
*Boesenbergia rotunda* extract and panduratin A reduced HNF1α expression at both mRNA and protein levels. imHC cells were transfected with 0–2 µg of pHBV 1.3-mer WT. On day 3, HNF1α (**A**) and HNF4α (**B**) mRNA levels were measured by real-time qPCR. The mRNA expression of liver-enriched transcription factors, including HNF1α, HNF4α, PPARα, and C/EBPα, was analyzed after treatment with 0–10 µM panduratin A (**C**). The effects of *B. rotunda* extract and panduratin A on HNF1α (**D**) and HNF4α (**E**) protein expression were assessed by Western Blot. HBV-infected d-imHC cells were treated with 0–1.25 µM panduratin A, and HNF1α and HNF4α protein levels were detected by Western Blot (**F**). Western Blot quantification was performed using ImageJ software, with normalization to GAPDH

The reduction of HBV promoter activity by *B. rotunda* extract contributes to its anti-HBV effects. HBV promoter activity is regulated by hepatocyte-enriched transcription factors, including HNF1α, HNF4α, FOXA2, HNF6, and PGC1α. Among these, we focused on HNF1α and HNF4α, as they predominantly regulate preS1, preS2, and core promoter activity. To determine whether HBV production affects HNF1α and HNF4α expression, the pHBV 1.3-mer WT plasmid (0.2–2 µg/10⁶ cells) was transfected into imHC cells for three days. Real-time qPCR analysis showed that HBV production did not alter HNF1α or HNF4α expression levels (Fig. 3A, B). Next, we examined the effect of panduratin A on liver-enriched transcription factors, including HNF1α, HNF4α, PPARα, and C/EBPα. The results demonstrated that treatment with 2.5–10 µM panduratin A significantly downregulated HNF1α expression by 30–70% in a dose-dependent manner, while HNF4α expression was slightly reduced by 25%. However, panduratin A did not affect PPARα or C/EBPα mRNA levels (Fig. 3C). Western Blot analysis further confirmed that treatment with 12.5–50 µg/mL *B. rotunda* extract reduced HNF1α protein levels by 20–25%, while HNF4α protein levels were slightly increased (Fig. 3D). Similarly, treatment with purified panduratin A (1.25–10 µM) led to a 25–50% decrease in HNF1α protein levels and a slight increase in HNF4α protein levels (Fig. 3E). To verify that the downregulation of HNF1α was mediated by panduratin A rather than induced by HBV infection, infected d-imHC cells were treated with 0–10 µM panduratin A, and HNF1α and HNF4α protein levels were measured. The results indicated that HBV infection itself did not alter HNF1α or HNF4α protein levels. Instead, the downregulation of HNF1α was specifically initiated by panduratin A treatment (Fig. 3F). These findings suggest that panduratin A downregulates HNF1α, thereby modulating HBV transcription, which may contribute to its anti-HBV activity.

### Panduratin A required HNF1α expression to exhibit anti-HBV activity

**Fig. 4:**
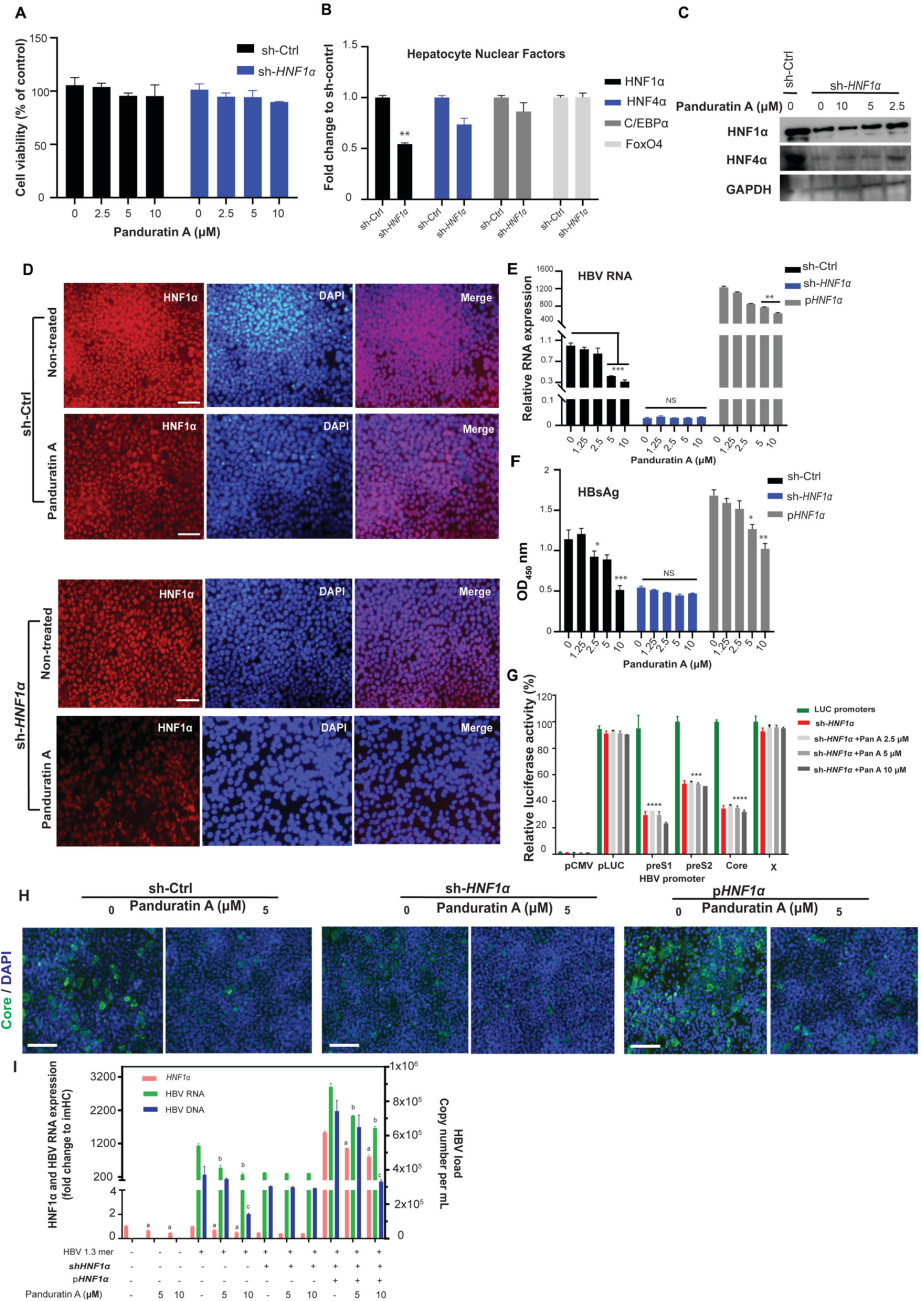
The anti-HBV activity of panduratin A is mediated through HNF1α and is impaired by HNF1α knockdown but restored by HNF1α rescue. imHC cells were transfected with sh-Ctrl, sh-HNF1α or HNF1α plasmids for 4 h before treatment with 0–10 µM panduratin A. On day 3, cell viability was assessed using the MTT assay (**A**). The mRNA expression of liver-enriched transcription factors (HNF1α, HNF4α, C/EBPα, and FoxO4) was analyzed in sh-Ctrl and HNF1α-knockdown imHCs (**B**). HNF1α-knockdown imHCs were treated with panduratin A, and the protein levels of HNF1α, HNF4α, and GAPDH were detected by Western Blot (**C**). Nuclear localization of HNF1α was analyzed in imHCs transfected with sh-HNF1α or sh-Ctrl following treatment with 5 µM panduratin A or 0.1% DMSO. Scale bar, 50 µm (**D**). HBV RNA levels were measured in infected imHCs transfected with sh-Ctrl, sh-HNF1α, or an HNF1α expression plasmid following treatment with 0–10 µM panduratin A (**E**), and HBsAg levels were quantified by ELISA (**F**). HBV promoter activities were evaluated in HNF1α-knockdown cells with or without panduratin A treatment; pCMV and pLuc served as negative and positive controls, respectively (**G**). Immunofluorescence analysis detected HBV core protein (green) and nuclei (DAPI, blue) in sh-HNF1α and HNF1α-transfected imHCs with or without 5 µM panduratin A treatment (**H**). HNF1α expression, HBV RNA levels, and viral load were measured in wild-type, HNF1α-knockdown, and HNF1α-knockdown cells with ectopic HNF1α expression, with or without panduratin A treatment (**I**). Scale bar, 50 µm. Data were presented as mean ± SD. * or a, ** or b, *** or c, and **** or d indicate statistical significance at p value < 0.05, < 0.01, < 0.001, and < 0.0001, respectively

To confirm the critical role of HNF1α in the anti-HBV activity of panduratin A, HNF1α expression was either silenced or ectopically expressed in hepatocytes using HNF1α-shRNA plasmid (sc-35567-SH, Santa Cruz Biotechnology) or an HNF1α plasmid (Addgene plasmid #31104), respectively, to assess the loss- and gain-of-function effects. Cell viability assays indicated no cytotoxicity in HNF1α-knockdown imHC cells treated with 0–10 µM panduratin A (Fig. 4A). HNF1α knockdown reduced HNF1α mRNA expression by approximately 50% and slightly decreased HNF4α mRNA levels by 20%, while the expression of C/EBPα and FoxO4 remained unchanged (Fig. 4B). Western Blot analysis confirmed a 50% reduction in HNF1α protein following shRNA-mediated knockdown compared to the control. Treatment with 2.5–10 µM panduratin A for three days further reduced HNF1α protein levels by 40–60%, without affecting HNF4α protein levels (Fig. 4C and Fig. S1H). Immunofluorescence staining revealed reduced nuclear localization of HNF1α in shRNA control imHC cells treated with 5 µM panduratin A (Fig. 4D, upper panel). The combination of HNF1α knockdown and 5 µM panduratin A significantly diminished nuclear HNF1α expression in hepatocytes (Fig. 4D, lower panel).

For the loss-of-function analysis, HNF1α knockdown alone decreased HBV RNA levels and HBsAg secretion by over 90% and 50%, respectively, thereby significantly impairing the dose-dependent inhibitory effects of panduratin A on HBV RNA transcription (Fig. 4E) and HBsAg secretion (Fig. 4F). Conversely, in the gain-of-function setting, ectopic expression of HNF1α resulted in a > 1200-fold increase in HBV RNA and a 1.75-fold increase in HBsAg secretion compared to mock transfection, thereby enhancing the dose-dependent inhibitory effect of panduratin A on both HBV RNA and HBsAg levels (Fig. 4E, F).

Further investigation of HNF1α function via HBV promoter activity assays demonstrated that HNF1α knockdown reduced preS1, preS2, and core promoter activities by 70%, 60%, and 40%, respectively. Treatment of HNF1α-knockdown imHC cells with 2.5–10 µM panduratin A further decreased only preS1 promoter activity by an additional 2–5% (Fig. 4G). HBV infectivity, assessed by immunofluorescence staining of HBcAg, was reduced by more than 90% following treatment with 5 µM panduratin A; however, this antiviral effect was markedly diminished in HNF1α-knockdown cells and enhanced in cells overexpressing HNF1α (Fig. 4H).

To evaluate the rescue effect, wild-type HBV 1.3-mer was transfected into wild-type, HNF1α-knockdown, and HNF1α-knockdown cells with ectopic HNF1α expression. In wild-type imHC cells, 10 µM panduratin A reduced HBV viral load by 50%. This antiviral activity was impaired in HNF1α-knockdown cells. Notably, ectopic re-expression of HNF1α in knockdown cells restored HBV RNA and viral load, and reinstated the antiviral efficacy of panduratin A, with 10 µM panduratin A again reducing viral load by approximately 50%, as observed in wild-type imHC cells (Fig. 4I).

Overall, these findings demonstrate that HNF1α knockdown markedly attenuated the inhibitory effects of panduratin A on HBV replication, HBsAg secretion, and viral infectivity, indicating that the anti-HBV activity of panduratin A is critically dependent on HNF1α function.

### Combinations of panduratin A with GS-5801 (KDM5 inhibitor) and NVR-3778 (capsid assembly modulator) exhibited synergistic anti-HBV effects

**Fig. 5:**
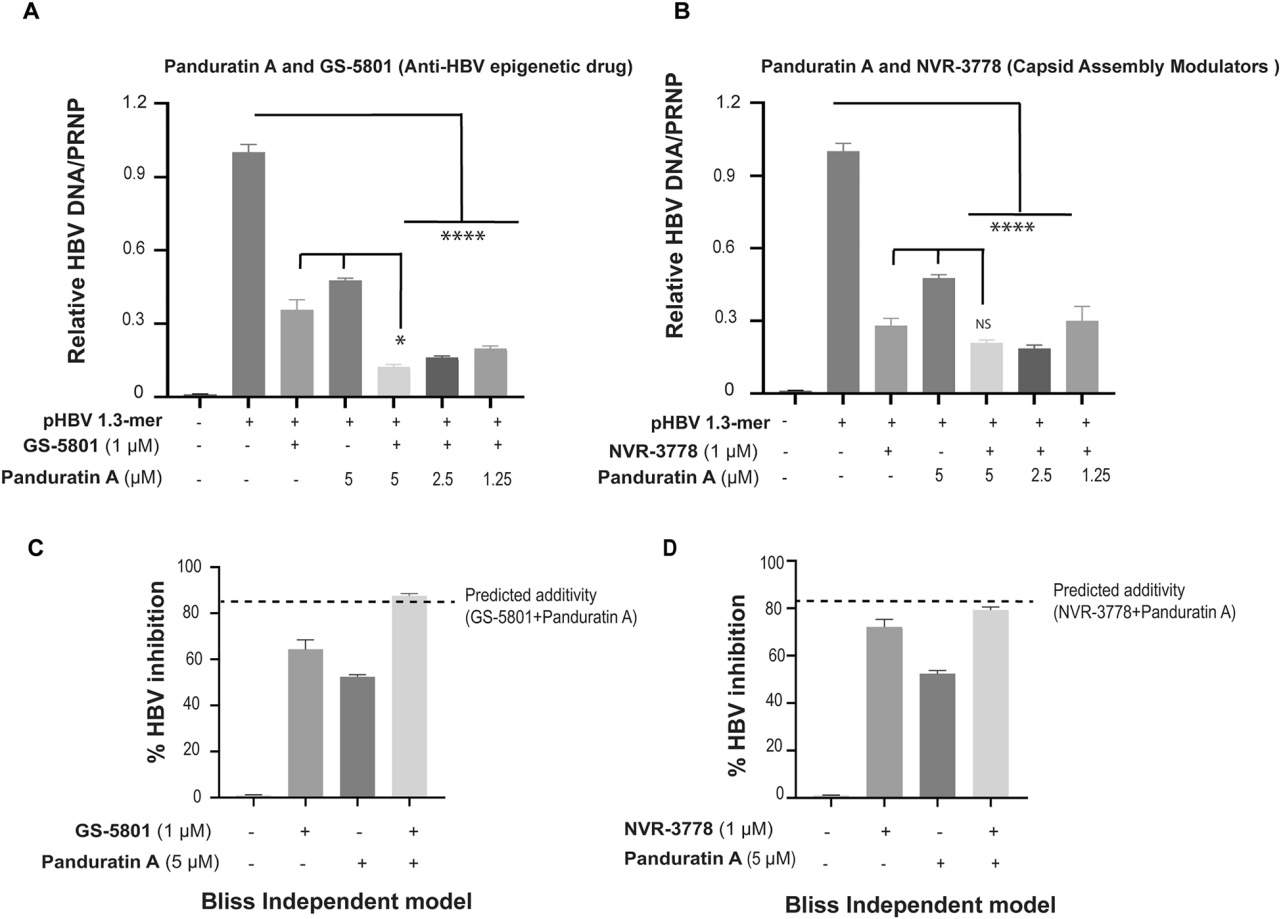
Combined effects of panduratin A with a KDM5 inhibitor or a capsid assembly modulator on HBV DNA levels. imHC cells were transfected with the pHBV 1.3-mer WT for 4 h before treatment with the indicated compounds. HBV DNA levels in transfected imHCs were measured following treatment with panduratin A alone or in combination with GS-5801 (a KDM5 inhibitor) (**A**) or NVR-3778 (a capsid assembly modulator) (**B**). The Bliss Independence model was used to predict the combinatory effects of GS-5801 (**C**) and NVR-3778 (**D**). Data were presented as mean ± SD. *, **, ***, and **** indicate statistical significance with p values of < 0.05, < 0.01, < 0.001, and < 0.0001, respectively

Our study indicated that panduratin A inhibited HBV transcription by modulating HBV promoters. To evaluate its antiviral efficacy, imHC cells were transfected with pHBV 1.3-mer WT for 4 h and subsequently treated with either 1.25–5 µM panduratin A as monotherapy, or in combination with 1 µM GS-5801 (a KDM5 inhibitor), or with 1 µM NVR-3778 (a capsid assembly modulator). As monotherapies, 1 µM GS-5801 and 5 µM panduratin A decreased HBV DNA levels by 60% and 50%, respectively. Notably, combining GS-5801 with 1.25–5 µM panduratin A resulted in a significant decrease in HBV DNA levels by 80–87% (Fig. 5A). Similarly, co-treatment with NVR-3778 and 1.25–5 µM panduratin A decreased HBV DNA levels by 70–82% (Fig. 5B).

Drug combination effects were further analyzed using the Bliss independence model. The results revealed that the combination of panduratin A and GS-5801 synergistically enhanced antiviral efficacy compared to monotherapy (Fig. 5C). In contrast, the combination of panduratin A and NVR-3778 exhibited an additive antiviral effect relative to monotherapy (Fig. 5D). These findings suggested that panduratin A, in combination with either GS-5801 or NVR-3778, enhanced anti-HBV activity through distinct mechanisms targeting different stages of the HBV life cycle.

### Liver spheroids for assessing the inhibitory effect of panduratin A

**Fig. 6:**
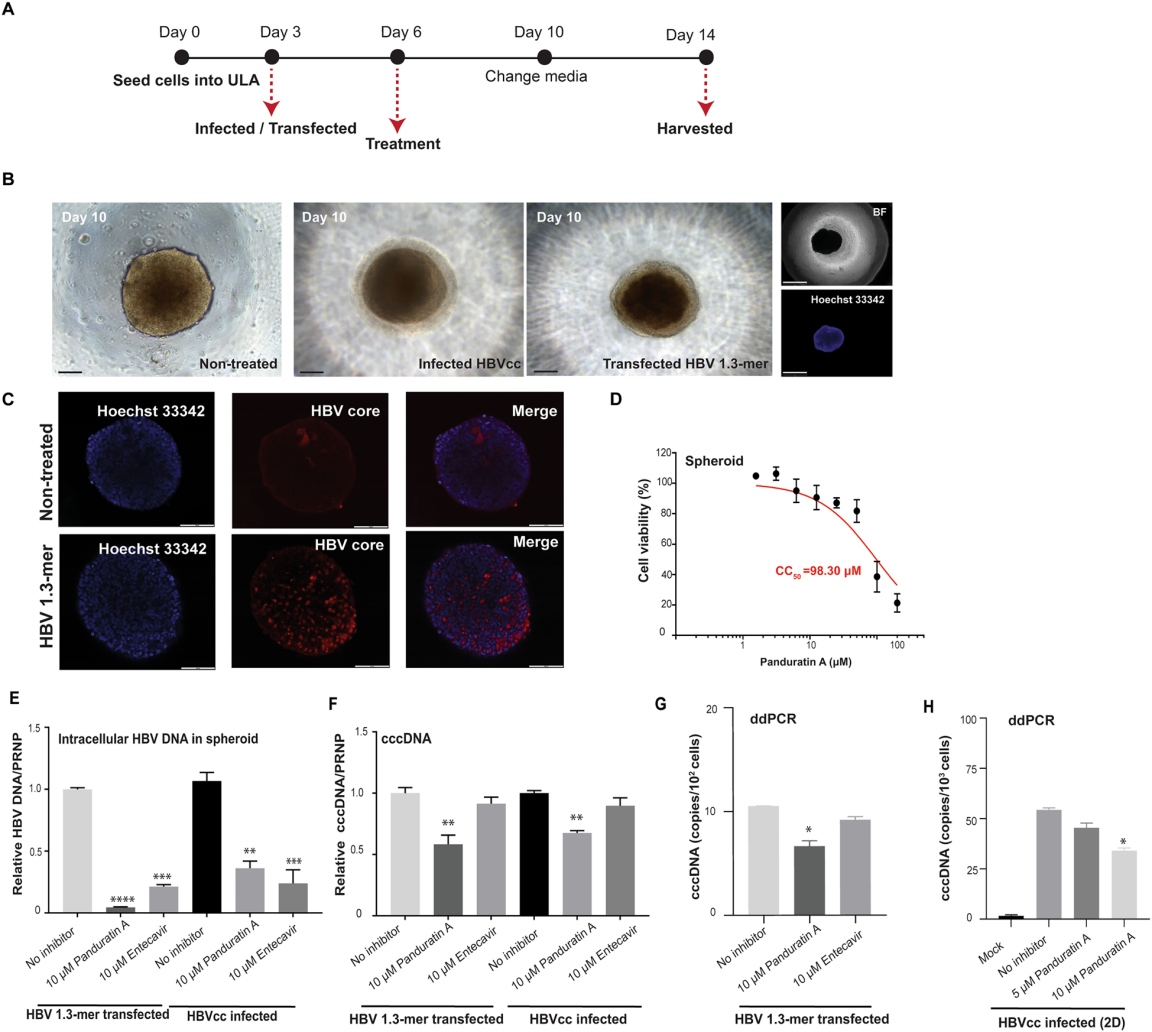
Enhanced anti-HBV activity and selectivity of panduratin A treatment in a 3D liver spheroid culture. Schematic diagram illustrating the 3D liver spheroid culture system for HBVcc infection and HBV 1.3-mer WT plasmid transfection (**A**). Morphology of liver spheroids in HBV-infected and HBV-transfected conditions compared to the mock control on day 10, observed under a 10 × inverted microscope. Scale bar, 20 µm (bright field, BF). Hoechst 33342 staining confirmed spheroid formation by labeling cell nuclei. Scale bar, 625 µm (Hoechst) (**B**). Immunofluorescence staining of Hoechst 33342 (blue) showing the entire spheroid structure, with HBV core (red) detected in 3D spheroids. Scale bar, 1 mm (**C**). Cytotoxicity of panduratin A was assessed in liver spheroids, and CC_50_ values were calculated (**D**). HBV DNA levels were measured in both infected and transfected imHC spheroids following treatment with panduratin A or entecavir (**E**). Relative cccDNA levels were also determined (**F**). cccDNA copy numbers were quantified in both spheroid (**G**) and two-dimensional (2D) HBV-infected imHCs (**H**) after panduratin A treatment. Data were presented as mean ± SD. *, **, ***, and **** indicate statistical significance with p values of < 0.05, < 0.01, < 0.001, and < 0.0001, respectively

Liver spheroids were generated by seeding imHC cells onto 96-well ultra-low attachment plates to mimic liver architecture. These hepatic spheroids were either infected with HBVcc at a MOI of 100 or transfected with pHBV 1.3-mer WT, followed by an 8-day treatment with the compounds to assess their anti-HBV activity (Fig. 6A). By day 10, liver spheroids reached a diameter of approximately 130–150 µm, with HBV production showing no impact on their size or morphology (Fig. 6B). The spheroids consistently supported HBV infection, as evidenced by the presence of HBcAg (Fig. 6C).

Cytotoxicity of panduratin A in liver spheroids was assessed, revealing a 50% cytotoxic concentration (CC_50_) of 98.30 µM (Fig. 6D). This CC_50_ was 6.2 times higher than that observed in monolayer imHC cells (15.91 µM), suggesting reduced cytotoxicity in the 3D spheroid model. In HBV-transfected spheroids, treatment with 10 µM panduratin A significantly reduced intracellular HBV DNA levels by 90%, surpassing the antiviral activity of 10 µM entecavir. However, in HBV-infected spheroids, 10 µM panduratin A and 10 µM entecavir reduced intracellular HBV DNA levels by 60% and 75%, respectively (Fig. 6E). Additionally, panduratin A treatment reduced cccDNA levels by approximately 40% in pHBV 1.3-mer WT-transfected spheroids and by 25% in HBVcc-infected spheroids, whereas entecavir treatment showed no significant effect on cccDNA levels (Fig. 6F). Quantitative analysis using ddPCR with cccDNA-specific primers and probe (Fig. S2D) further confirmed the reduction in cccDNA copy numbers in both spheroid (Fig. 6G) and two-dimensional (2D) infected models (Fig. 6H).

These findings revealed that the antiviral activity of panduratin A was significantly enhanced in liver spheroids, attributed to its reduced cytotoxicity and improved efficacy within a more physiologically relevant model.

### Database prediction of HNF1α, HNF4α, PPARα, and C/EBPα interaction networks

**Fig. 7:**
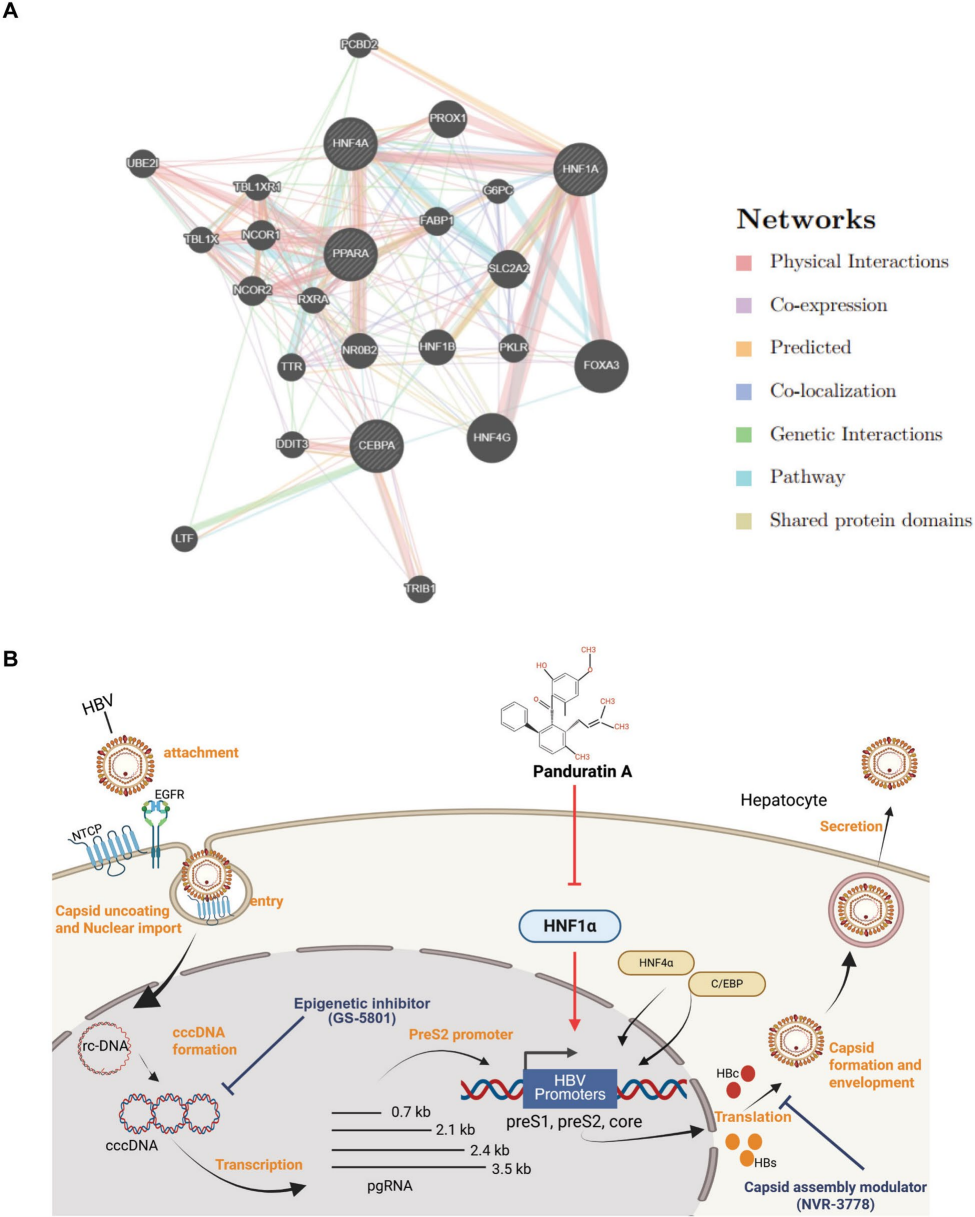
Interaction networks of HNF1α, HNF4α, PPARα, and C/EBPα based on GeneMANIA. Gene interaction analysis was conducted using GeneMANIA to explore potential mechanisms underlying the anti-HBV effects of panduratin A. The analysis visualizes interaction types (indicated by different colors), interaction strengths (represented by edge thickness), multiple edges between nodes, and protein scores (reflected by node sizes) (**A**). A schematic diagram illustrating the proposed mechanism by which panduratin A inhibits HBV replication in hepatocytes. Panduratin A suppresses HNF1α, which in turn regulates the HBV promoter. Created in BioRender, https://BioRender.com/q22t488 (**B**)

GeneMANIA was utilized to analyze the protein–protein interaction network involving HNF1α, HNF4α, PPARα, and C/EBPα, key regulators of HBV promoter activity [25]. The analysis identified 16 proteins associated with these transcription factors, with 77.67% of interactions categorized as physical interactions and 8.91% as co-expression relationships (Fig. 7A). Consistent with previous studies and our findings, these results underscore the pivotal role of hepatocyte nuclear factors (HNFs), PPARα, and C/EBPα in regulating HBV promoters (Fig. 7B). Based on this evidence, we propose that panduratin A suppresses HBV transcription by modulating HNF1α and HNF4α activity. This mechanism positions panduratin A as a promising therapeutic candidate for HBV treatment, either as a standalone therapy or in combination with nucleos(t)ide analogs (NAs).

## Discussion

*Boesenbergia rotunda*, commonly known as fingerroot, is a member of the Zingiberaceae family, which includes ginger. Crude extract derived from fingerroot rhizomes is renowned for their diverse pharmacological properties, such as anti-dengue, anti-HIV-1 protease, anti-inflammatory, antioxidant, anti-cancer, antibacterial, anti-aging, and anti-obesity properties [18, 26–37]. Our previous research identified anti-SARS-CoV-2 activity in *B. rotunda* extract and its compounds, panduratin A and pinostrobin, with the crude extract containing 6.042% (w/w) panduratin A [16]. The pharmacokinetics of panduratin A, including its oral bioavailability, tissue distribution, metabolism, and excretion, were evaluated in healthy rats [38]. Molecular docking suggested panduratin A as an inhibitor of several SARS-CoV-2 proteins included main protease (Mpro), papain-like protease (PLpro), receptor binding domain (RBD), RNA-dependent RNA polymerase (RdRp) and 2'-O-methyltransferase (MTase) [39]. Additionally, panduratin A demonstrated hepatoprotective effects in thioacetamide-induced liver damage models [20, 40]. However, its antiviral activity against hepatitis viruses, especially hepatitis B, had not been explored.

Here, we showed that *Boesenbergia rotunda* extract, panduratin A, and pinostrobin inhibited HBV propagation in infected imHC cells. These effects were dose- and time-dependent, significantly reducing intracellular HBV DNA, viral load, HBsAg, and HBeAg, outperforming entecavir. Panduratin A and pinostrobin displayed minimal cytotoxicity on imHC cells, with CC_50_ values of 15.91 µM and > 100 µM, respectively, with selectivity indexes (SI) of 2.94 and > 10. Panduratin A also reduced HBV pgRNA levels, prompting us to examine its impact on HBV promoter activity [41]. The crude extract and panduratin A downregulated preS1, preS2, and preC promoter activities in a dose-dependent manner, consequently reducing the corresponding mRNA and protein levels. The preS1 promoter regulates the transcription of large (L) mRNA (2.4 kb), while preS2 controls the transcription of small (S) and medium (M) mRNAs (2.1 kb). The preC promoter (3.5 kb) initiates the transcription of pre-genomic (pg) RNAs, including HBe and polymerase genes [42, 43]. This transcriptional activity is essential for the synthesis of HBV structural and non-structural proteins, which are crucial for the HBV life cycle [44].

Liver-enriched transcription factors have been known to regulate HBV promoters, enhancing viral transcription [45, 46]. Among these, hepatocyte nuclear factors (HNFs) have been identified as key modulators of HBV promoters by activating HBV transcription [47]. Specifically, HNF1α promotes HBV transcription by binding to the HBV enhancer/promoter regions of preS1 and Enh II B2, thereby activating core promoters. HNF1α and HNF4α have emerged as pivotal transcriptional regulators that activate HBV gene expression by binding to key enhancer and promoter regions [48–51]. Overexpression of HNF4α has been shown to increase the activity of preS1, preS2, and core promoters [52–54]. In our study, HNF1α overexpression elevated HBV RNA levels and HBsAg secretion, and further enhanced the antiviral effect of panduratin A. Both HNF1α and HNF4α were associated with the ERK-HNF1α-C/EBPα-HNF4α axis [55]. While HBV replication did not affect the expression levels of HNF1α and HNF4α, both mRNA and protein levels of these factors were reduced by *B. rotunda* extract and panduratin A. To elucidate panduratin A's mechanism, shRNA-mediated HNF1α knockdown in imHC cells was performed. HNF1α protein level was reduced by 50% in sh-HNF1α imHC cells, and this inhibitory effect was further enhanced by panduratin A. HNF1α knockdown reduced HBV DNA, HBsAg and HBcAg levels by over 90%, 50% and 90%, respectively. Interestingly, this knockdown also attenuated the anti-HBV activity of panduratin A, paralleling findings with other antiviral agents like saikosaponin C [56] and baicalin [57]. Baicalin (BA), a flavonoid derived from *Scutellaria baicalensis*, has been shown to inhibit HBV RNA in HepG2.2.15 cells by downregulating the HBV replication-dependent host factors HNF1α and HNF4α [58]. The role of the liver-specific HNF4α-HNF1α axis was demonstrated through shRNA knockdown, which clarified BA's anti-HBV activity. In our study, HNF1α knockdown impaired the inhibitory effects of panduratin A on HBV RNA, HBsAg secretion, and core protein expression. Restoration of HNF1α through ectopic expression reversed this impairment and reinstated the antiviral activity. These results suggest that the ERK–HNF1α–C/EBPα–HNF4α regulatory axis plays a critical role in the antiviral mechanism of panduratin A.

Combination therapies are pivotal for managing chronic viral infections, as seen in HIV and HCV treatments [59, 60]. For HBV, combination therapy aims to achieve long-term viral suppression and reduce viral load while minimizing adverse effects and drug resistance [61]. Combining panduratin A with GS-5801 (KDM5 inhibitor) or NVR-3778 (capsid assembly modulator) produced additive or synergistic effects, independently targeting HBV promoters and cccDNA. The combination of panduratin A and GS-5801 significantly inhibited HBV replication, confirmed by Bliss model analysis [62]. Similarly, the addition of NVR-3778 enhanced panduratin A's antiviral activity by inhibition of HBV capsid assembly, aligning with previous reports describing additive or synergistic effects when NVR-3778 was combined with nucleos(t)ide analogs (LMV, TFV, or ETV), RNase H agonists, or other inhibitors [63, 64]. These findings position *B. rotunda* extract and panduratin A as promising adjunct therapies for chronic hepatitis B infection.

To address the limitations of 2D cell culture models, which lack liver architecture and adequate biological barriers, we developed a 3D liver spheroid model for drug screening and HBV infection studies [65]. These spheroids, derived from imHC cells, supported HBVcc infection and HBV 1.3-mer WT transfection, consistently showing positive HBcAg expression. In this 3D culture, the CC_50_ value of panduratin A was six times higher than in 2D models, highlighting its safety owing to the liver-like architecture that improved metabolic functions [66]. Both HBV DNA and cccDNA responded to panduratin A and entecavir treatments, confirming the model's utility for evaluating antiviral activity. Like the long-term 3D cell culture model, de novo HBV-infected mouse-passaged (mp)PHH were responsive to a capsid assembly modulator, highlighting their potential as a drug-testing platform [67].

The interactions among liver-specific transcription factors, the HBV receptor, and HBV were investigated. BEL7404 cells express HNF1α but have insufficient levels of HNF4α, rendering them resistant to HBV infection. However, ectopic expression of both HNF4α and NTCP restored their capacity to support HBV replication and production [68]. The neddylation inhibitor MLN4924 has been reported to exert anti-HBV activity by modulating the ERK–HNF1α–C/EBPα–HNF4α signaling axis [55]. The activities of HNF4α and HNF1α are crucial for HBV replication and are closely associated with hepatocyte differentiation. Notably, HNF1α regulates HBV replication through its control of HNF4α expression [69]. Gene network analysis in this study revealed that panduratin A disrupted the interactions between HNF1α and other key transcription factors, including C/EBPα, PPARα, and FOXA3 (HNF-3γ).

Panduratin A treatment or HNF1α knockdown via shRNA disrupted these interactions, highlighting the HNF1α axis as a potential mechanism driving the anti-HBV activity of panduratin A. A critical factor in HBV persistence is the stability of cccDNA within infected hepatocytes. This study suggests that *B. rotunda* extract and panduratin A may destabilize cccDNA and suppress its transcriptional activity, thereby limiting the viral template reservoir for replication. Notably, the suppression of HNF1α, a crucial transcription factor for HBV replication, underscores the therapeutic potential of *B. rotunda* extract. By disrupting the interaction between HNF1α and HBV regulatory elements, these compounds effectively suppressed viral transcription and replication.

In summary, our findings demonstrated that *B. rotunda* extract and its bioactive compound, panduratin A, exerted significant inhibitory effects on the HBV life cycle, particularly by suppressing the maintenance and transcriptional activity of HBV DNA and cccDNA. These results indicated that both compounds effectively inhibited HBV replication and downregulated key viral promoters. Specifically, *B. rotunda* extract and panduratin A suppressed the activity of HBV promoters (preS1, preS2, and core), which were enhanced by hepatocyte-enriched nuclear factors such as HNF1α. Notably, the inhibition of HNF1α by panduratin A suggested a targeted mechanism underlying its antiviral effects.

## Supplementary Information

Supplementary Material 1.

Supplementary Material 2 .

Supplementary Material 3.

## Data Availability

No datasets were generated or analysed during the current study.

## Abbreviations

C/EBPα: CCAAT Enhancer-Binding Protein Alpha
cccDNA: Covalently Closed Circular DNA
DAA: Direct-Acting Antiviral
DMSO: Dimethyl Sulfoxide
ELISA: Enzyme-Linked Immunosorbent Assay
ETV: Entecavir
FBS: Fetal Bovine Serum
FoxO4: Forkhead Box O4
GAPDH: Glyceraldehyde-3-Phosphate Dehydrogenase
HBsAg: Hepatitis B Surface Antigen
HBeAg: Hepatitis B e Antigen
HBV: Hepatitis B Virus
HNF1α: Hepatocyte Nuclear Factor 1 Alpha
HNF4α: Hepatocyte Nuclear Factor 4 Alpha
HRP: Horseradish Peroxidase
imHC: Immortalized Human Hepatocyte Cells
LMV: Lamivudine
MOI: Multiplicity of Infection
MTT: 3-(4,5-Dimethylthiazol-2-yl)-2,5-Diphenyltetrazolium Bromide (MTT Assay)
PBS: Phosphate-Buffered Saline
PPARα: Peroxisome Proliferator-Activated Receptor Alpha
PVDF: Polyvinylidene Difluoride
qPCR: Quantitative Polymerase Chain Reaction
*B. Rotunda*: *Boesenbergia rotunda*
SDS-PAGE: Sodium Dodecyl Sulfate–Polyacrylamide Gel Electrophoresis
shRNA: Short Hairpin RNA
TAF: Tenofovir Alafenamide
TDF: Tenofovir Disoproxil Fumarate
WT: Wild-Type
