# Supplementary material for: Panduratin A from Boesenbergia rotunda suppresses hepatitis B virus by targeting HNF1α and synergizing with antiviral agents
Source: Chin Med. 2026 Jan 7;21:10. doi: 10.1186/s13020-025-01285-w (PMC12777152; doi:10.1186/s13020-025-01285-w)
Supplement: Supplementary file 1 — Supplementary Material 1. [file 13020_2025_1285_MOESM1_ESM.docx]

**Supplementary file**

**Fig. S1.** **Cytotoxicity, antiviral activity and promoter activity modulation of *B. rotunda* extract, panduratin A, and pinostrobin**. Chemical structures of panduratin A and pinostrobin, flavonoid-derived compounds from *B. rotunda* extract, with functional groups highlighted in red (A). Cytotoxic effects of *B. rotunda* extract, panduratin A, and pinostrobin in imHC cells were assessed by MTT assay, with CC₅₀ values of 72.30 μg/mL, 15.91 μM, and >100 μM, respectively (C–E). Effects of 0–25 μM entecavir on HBV promoter activity were evaluated by luciferase assay (F). Immunofluorescence staining of HBV core (upper) and preS2 (lower) proteins in HBV-infected cells treated with 5 μM entecavir versus control (G). Relative protein expression of HNF1α and HNF4α in sh-HNF1α knockdown imHC cells treated with panduratin A (H).

**Fig. S2. Panduratin A reduces HBx RNA and protein expression and illustrates cccDNA quantification strategy.** Quantitative analysis of HBx RNA expression in both infected imHC (A) and HBx-imHC cells with ectopic HBx expression (B) after treatment with 0–10 µM panduratin A for three days, as determined by real-time qPCR. Flow cytometry analysis showing the inhibitory effect of panduratin A on HBx protein expression (C). Schematic representation of the Hepatitis B Virus (HBV) relaxed circular DNA (rcDNA) and covalently closed circular DNA (cccDNA) structures (D). The locations of the two direct repeats (DRs) at nucleotides (nt) 1826 and 1592 are indicated. cccDNA-specific primers, designed to span the unique gap-closure region, were used for quantitative analysis by droplet digital PCR (ddPCR). Right: representative two-dimensional droplet scatter plots showing quantification of cccDNA and the reference gene (RPP30) from the infected-imHC model. Data are presented as mean ± SD. *, **, ***, and **** indicate statistical significance with p < 0.05, p < 0.01, p < 0.001, and p < 0.0001, respectively.

**Table S1** Primer sets for host gene expression and HBV detection used in real-time qPCR and ddPCR

| **Detection** | **Sequences of primers** | **Annealing Temperature (°C)** | **Amplicon size (bp)** |
| --- | --- | --- | --- |
| HBV DNA | Forward: 5’-GTTGCCCGTTTGTCCTCTAATTC-3’ | 60 | 100 |
|  | Reverse: 5’-GGAGGGATACATAGAGGTTCCTTGA-3’ |  |  |
| PRNP | Forward: 5’-GACCAATTTATGCCTACAGC-3’ | 60 | 112 |
|  | Reverse: 5’-TTTATGCCTACAGCCTCCTA-3’ |  |  |
| HBV pgRNA | Forward: 5’-TGTTCAAGCCTCCAAGCT-3’ | 60 | 114 |
|  | Reverse: 5’-GGAAAGAAGTCAGAAGGCAA-3’ |  |  |
| HBV RNA | Forward: 5’-GCACTTCGCTTCACCTCTGC-3’ | 60 | 120 |
|  | Reverse: 5’-CTCAAGGTCGGTCGTTGACA-3’ |  |  |
| GAPDH | Forward: 5’-GAAATCCCATCACCATCTTCC-3’ | 60 | 124 |
|  | Reverse: 5’-AAATGAGCCCCAGCCTTCTC-3’ |  |  |
| HNF1α | Forward: 5’-TACACCTGGTACGTCCGCAA-3’ | 60 | 109 |
|  | Reverse: 5’-CACTTGAAACGGTTCCTCCG-3’ |  |  |
| HNF4α | Forward: 5’-GCCTACCTCAAAGCCATCAT-3’ | 60 | 275 |
|  | Reverse: 5’-GACCCTCCCAGCAGCATCTC-3’ |  |  |
| PPARα | Forward: 5’-GCCTGTCTGTCGGGATGT-3’ | 60 | 158 |
|  | Reverse: 5’-GGCTTCGTGGATTCTCTTG-3’ |  |  |
| C/EBPα | Forward: 5’-AGCCTTGTTTGTACTGTATG-3’ | 60 | 200 |
|  | Reverse: 5’-AAAATGGTGGTTTAGCAGAG-3’ |  |  |
| FoxO4 | Forward: 5’-ACGAGTGGATGGTCCGTACT-3’ | 60 | 86 |
|  | Reverse: 5’-GTGGCGGATCGAGTTCTTC-3’ |  |  |
| cccDNA | Forward: 5’-GACTCTCTCGTCCCCTTCTC-3’ | 60 | 579 |
|  | Reverse: 5’-ATGGTGAGGTGAACAATGCT-3’ |  |  |
| qHBx | Forward: 5’-GCTGCTAGGTTGTACTGCCA-3’ | 60 | 249 |
|  | Reverse: 5’-TTCACGGTGGTCTCCATGC-3’ |  |  |
| cccDNA (ddPCR) | Forward: 5’-CTTCTCATCTGCCGGACC-3′ | 58 | 323 |
|  | Reverse: 5’-CACAGCTTGGAGGCTTGA-3′ |  |  |
|  | Probe: 5’-AGGCTGTAGGCATAAATTGGTCT-3′ |  |  |
| RPP30 (ddPCR) | Forward: 5’-CTGCTTTTGGAATTATCTCTAC-3′ | 58 | 74 |
|  | Reverse: 5’-GGAAGCTGGAAGACAATC-3′ |  |  |
|  | Probe: 5’-AACCTCGGCCATCAGAAGGAGAT-3 |  |  |
