# Supplementary figures and images for: Panduratin A from Boesenbergia rotunda suppresses hepatitis B virus by targeting HNF1α and synergizing with antiviral agents
Source: Chin Med. 2026 Jan 7;21:10. doi: 10.1186/s13020-025-01285-w (PMC12777152; doi:10.1186/s13020-025-01285-w)

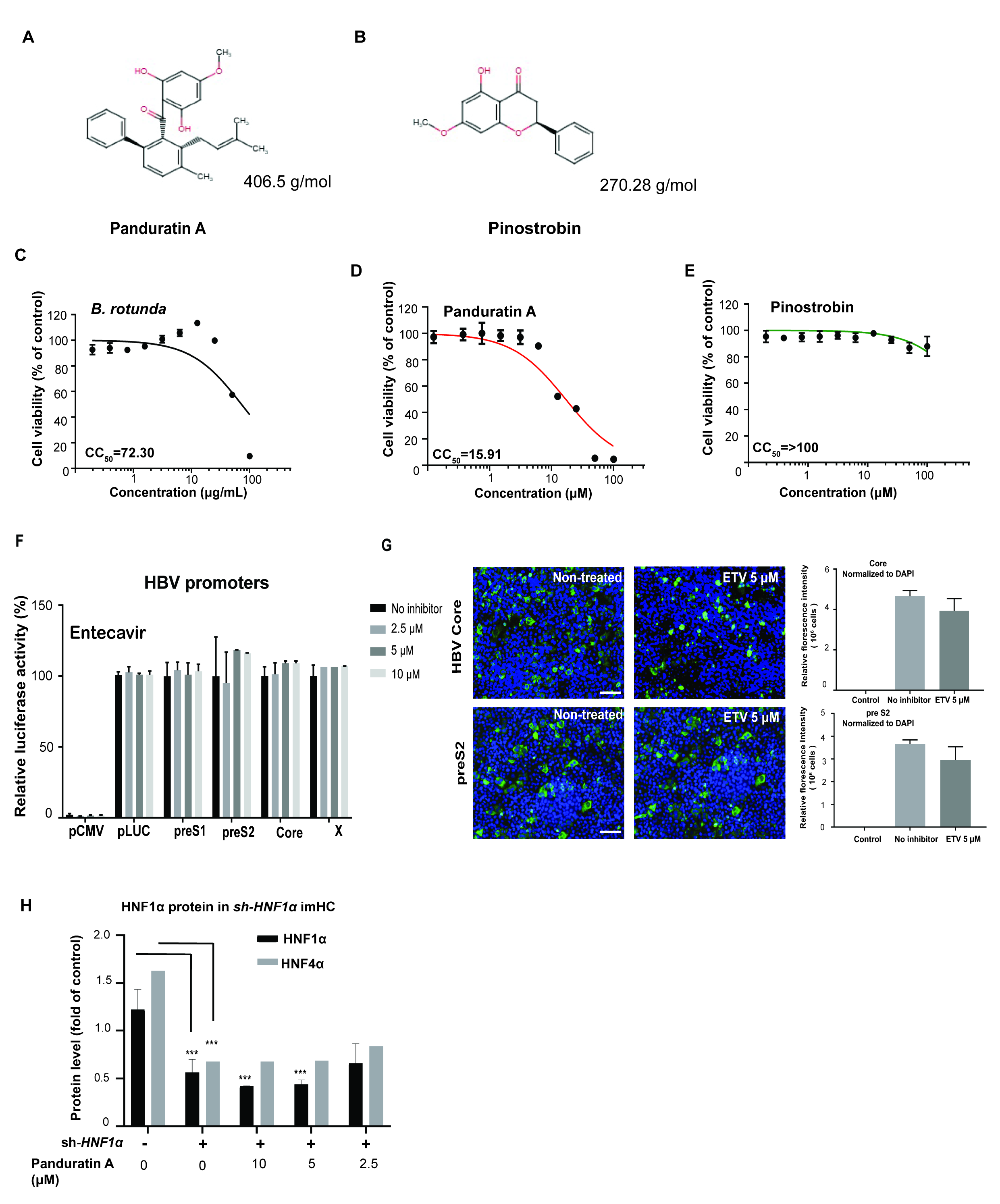

Supplement: Supplementary file 2 — Supplementary Material 2 . [file 13020_2025_1285_MOESM2_ESM.tif]

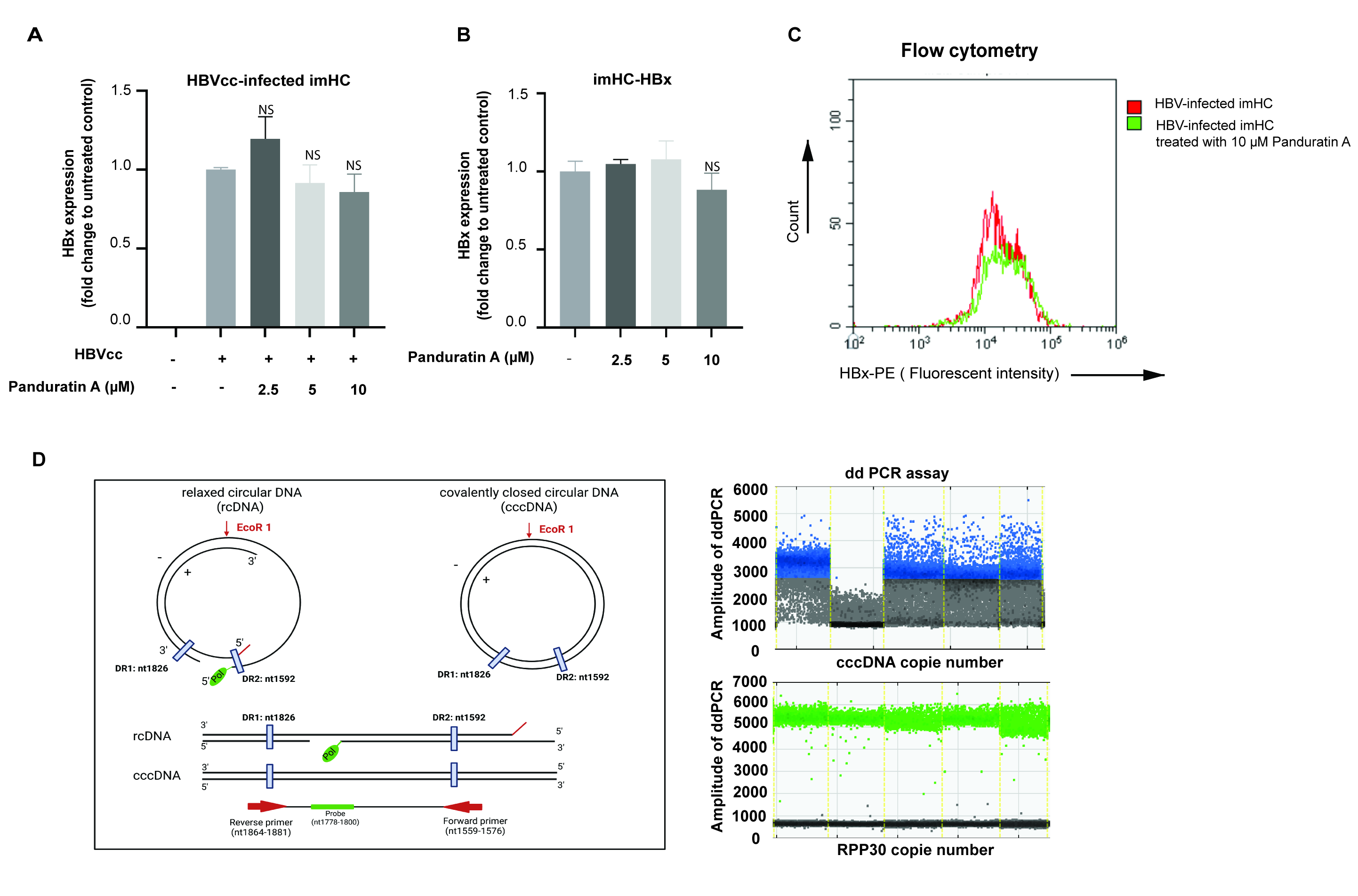

Supplement: Supplementary file 3 — Supplementary Material 3. [file 13020_2025_1285_MOESM3_ESM.tif]
